# *N*′-[(*E*)-(5-Bromo-2-hydroxy­phen­yl)(phen­yl)methyl­idene]-4-chloro­benzo­hydrazide

**DOI:** 10.1107/S1600536809027949

**Published:** 2009-07-22

**Authors:** Chang-Zheng Zheng, Chang-You Ji, Xiu-Li Chang

**Affiliations:** a College of Environment and Chemical Engineering, Xi’an Polytechnic University, 710048 Xi’an, Shaanxi, People’s Republic of China; b Department of Material Science and Chemical Engineering, Sichuan University of Science and Engineering, 643000 Zigong, Sichuan, People’s Republic of China

## Abstract

The Schiff base, C_20_H_14_BrClN_2_O_2_, displays a *trans* conformation with respect to the C=N double bond. The aromatic rings at either end of the –C(=O)–NH–N=C– fragment are nearly parallel [dihedral angle = 3.4 (5)°]. The hydr­oxy group forms an intra­molecular hydrogen bond to the imino N atom.

## Related literature

The chemistry of aroylhydrazones continues to attract much attention due to their ability to coordinate to metal ions (Singh *et al.*, 1982 ▶; Salem, 1998 ▶) and their biological activity (Singh *et al.*, 1982 ▶; Carcelli *et al.*, 1995 ▶).
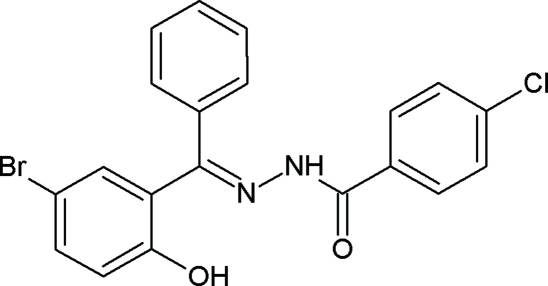


## Experimental

### 

#### Crystal data

C_20_H_14_BrClN_2_O_2_
*M* *_r_* = 429.69
Triclinic, 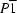

*a* = 7.3664 (8) Å
*b* = 10.6894 (11) Å
*c* = 12.3029 (14) Å
α = 71.976 (2)°
β = 82.228 (2)°
γ = 85.466 (2)°
*V* = 912.05 (17) Å^3^
*Z* = 2
Mo *K*α radiation
μ = 2.42 mm^−1^
*T* = 273 K
0.20 × 0.16 × 0.13 mm

#### Data collection

Bruker SMART area-detector diffractometer
Absorption correction: multi-scan (*SADABS*; Sheldrick, 1996 ▶) *T*_min_ = 0.644, *T*_max_ = 0.744
4841 measured reflections
3189 independent reflections
2467 reflections with *I* > 2σ(*I*)
*R*_int_ = 0.015

#### Refinement

*R*[*F* ^2^ > 2σ(*F* ^2^)] = 0.034
*wR*(*F* ^2^) = 0.085
*S* = 1.04
3189 reflections
237 parameters
H-atom parameters constrained
Δρ_max_ = 0.32 e Å^−3^
Δρ_min_ = −0.44 e Å^−3^

### 

Data collection: *SMART* (Bruker, 1996 ▶); cell refinement: *SAINT* (Bruker, 1996 ▶); data reduction: *SAINT*; program(s) used to solve structure: *SHELXS97* (Sheldrick, 2008 ▶); program(s) used to refine structure: *SHELXL97* (Sheldrick, 2008 ▶); molecular graphics: *SHELXTL* (Sheldrick, 2008 ▶); software used to prepare material for publication: *SHELXTL*.

## Supplementary Material

Crystal structure: contains datablocks I, global. DOI: 10.1107/S1600536809027949/ng2613sup1.cif

Structure factors: contains datablocks I. DOI: 10.1107/S1600536809027949/ng2613Isup2.hkl

Additional supplementary materials:  crystallographic information; 3D view; checkCIF report

## Figures and Tables

**Table 1:** Hydrogen-bond geometry (Å, °)

*D*—H⋯*A*	*D*—H	H⋯*A*	*D*⋯*A*	*D*—H⋯*A*
O1—H1⋯N1	0.82	1.84	2.554 (3)	145

**Fig. 1.**
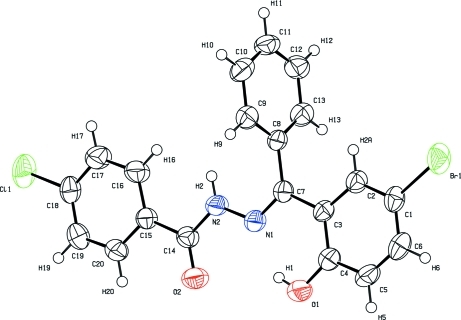
The molecular structure of compound (I), showing the atom-numbering scheme. Displacement ellipsoids are drawn at the 30% probability level.

## supplementary crystallographic information

### Comment

The chemistry of aroylhydrazones continues to attract much attention due to their coordination ability to metal ions (Singh *et al.*, 1982; Salem, 1998) and their biological activity (Singh *et al.*, 1982; Carcelli *et al.*, 1995). As an extension of work on the structural characterization of aroylhydrazone derivatives,the title compound, (I),was synthesized and its crystal structure is reported here.

The title molecule displays a *trans* conformation with respect to the C7=N1 double bond (Fig. 1). The crystal structure is stabilized by intramolecular O—H···N hydrogen bonds (Table).

### Experimental

4-chlorobenzohydrazide(0.02 mol,3.42 g) was dissolved in anhydrous ethanol (50 ml), and 1-(5-bromo-2-hydroxyphenyl)ethanone (0.02 mol, 4.30 g) was added. The reaction mixture was refluxed for 6 h with stirring, then the resulting precipitate was collected by filtration, washed several times with ethanol and dried *in vacuo* (yield 85%). The compound (2.0 mmol,0.68 g) was dissolved in dimethylformamide (30 ml) and kept at room temperature for 30 d to obtain yellow single crystals suitable for X-ray diffraction.

### Refinement

All H atoms were positioned geometrically and treated as riding on their parent atoms,with C—H(aromatic) = 0.93 Å, O—H = 0.82 Å, and N—H = 0.86 Å and with *U*_iso_(H) =1.2*U*_eq_(C_aromatic_,*N*).

### Crystal data

**Table:**

C_20_H_14_BrClN_2_O_2_	*Z* = 2
*M**_r_* = 429.69	*F*(000) = 432
Triclinic, *P*1	*D*_x_ = 1.565 Mg m^−^^3^
Hall symbol: -P 1	Mo *K*α radiation, λ = 0.71073 Å
*a* = 7.3664 (8) Å	Cell parameters from 1806 reflections
*b* = 10.6894 (11) Å	θ = 2.8–25.3°
*c* = 12.3029 (14) Å	µ = 2.42 mm^−^^1^
α = 71.976 (2)°	*T* = 273 K
β = 82.228 (2)°	Block, yellow
γ = 85.466 (2)°	0.20 × 0.16 × 0.13 mm
*V* = 912.05 (17) Å^3^	

### Data collection

**Table:**

Bruker SMART area-detector diffractometer	3189 independent reflections
Radiation source: fine-focus sealed tube	2467 reflections with *I* > 2σ(*I*)
graphite	*R*_int_ = 0.015
φ and ω scans	θ_max_ = 25.1°, θ_min_ = 1.8°
Absorption correction: multi-scan (*SADABS*; Sheldrick, 1996)	*h* = −8→8
*T*_min_ = 0.644, *T*_max_ = 0.744	*k* = −8→12
4841 measured reflections	*l* = −14→14

### Refinement

**Table:**

Refinement on *F*^2^	Primary atom site location: structure-invariant direct methods
Least-squares matrix: full	Secondary atom site location: difference Fourier map
*R*[*F*^2^ > 2σ(*F*^2^)] = 0.034	Hydrogen site location: inferred from neighbouring sites
*wR*(*F*^2^) = 0.085	H-atom parameters constrained
*S* = 1.04	*w* = 1/[σ^2^(*F*_o_^2^) + (0.0366*P*)^2^ + 0.3768*P*] where *P* = (*F*_o_^2^ + 2*F*_c_^2^)/3
3189 reflections	(Δ/σ)_max_ = 0.001
237 parameters	Δρ_max_ = 0.32 e Å^−^^3^
0 restraints	Δρ_min_ = −0.43 e Å^−^^3^

### Special details

**Table:**

Geometry. All e.s.d.'s (except the e.s.d. in the dihedral angle between two l.s. planes) are estimated using the full covariance matrix. The cell e.s.d.'s are taken into account individually in the estimation of e.s.d.'s in distances, angles and torsion angles; correlations between e.s.d.'s in cell parameters are only used when they are defined by crystal symmetry. An approximate (isotropic) treatment of cell e.s.d.'s is used for estimating e.s.d.'s involving l.s. planes.
Refinement. Refinement of *F*^2^ against ALL reflections. The weighted *R*-factor *wR* and goodness of fit *S* are based on *F*^2^, conventional *R*-factors *R* are based on *F*, with *F* set to zero for negative *F*^2^. The threshold expression of *F*^2^ > σ(*F*^2^) is used only for calculating *R*-factors(gt) *etc*. and is not relevant to the choice of reflections for refinement. *R*-factors based on *F*^2^ are statistically about twice as large as those based on *F*, and *R*- factors based on ALL data will be even larger.

### Fractional atomic coordinates and isotropic or equivalent isotropic displacement parameters (Å^2^)

**Table:**

	*x*	*y*	*z*	*U*_iso_*/*U*_eq_	
Br1	0.36717 (5)	1.17236 (4)	1.00467 (3)	0.06975 (17)	
Cl1	0.08513 (13)	0.47987 (9)	0.23226 (8)	0.0729 (3)	
O1	0.3069 (3)	1.25145 (19)	0.50596 (17)	0.0627 (6)	
H1	0.2882	1.1830	0.4931	0.094*	
O2	0.2209 (3)	1.0374 (2)	0.33927 (17)	0.0587 (6)	
N1	0.2632 (3)	1.0045 (2)	0.55663 (18)	0.0454 (6)	
N2	0.2388 (3)	0.9007 (2)	0.51767 (19)	0.0480 (6)	
H2	0.2367	0.8215	0.5636	0.058*	
C1	0.3457 (4)	1.1936 (3)	0.8483 (2)	0.0465 (7)	
C2	0.3209 (4)	1.0858 (3)	0.8134 (2)	0.0432 (6)	
H2A	0.3141	1.0027	0.8673	0.052*	
C3	0.3060 (3)	1.0998 (2)	0.6988 (2)	0.0389 (6)	
C4	0.3189 (4)	1.2264 (3)	0.6188 (2)	0.0455 (7)	
C5	0.3465 (4)	1.3328 (3)	0.6566 (3)	0.0540 (8)	
H5	0.3564	1.4163	0.6036	0.065*	
C6	0.3595 (4)	1.3169 (3)	0.7695 (3)	0.0534 (8)	
H6	0.3775	1.3891	0.7932	0.064*	
C7	0.2801 (4)	0.9829 (3)	0.6636 (2)	0.0397 (6)	
C8	0.2759 (4)	0.8493 (2)	0.7500 (2)	0.0380 (6)	
C9	0.4302 (4)	0.7655 (3)	0.7577 (2)	0.0474 (7)	
H9	0.5367	0.7920	0.7083	0.057*	
C10	0.4261 (5)	0.6424 (3)	0.8389 (3)	0.0544 (8)	
H10	0.5296	0.5861	0.8433	0.065*	
C11	0.2707 (5)	0.6030 (3)	0.9127 (3)	0.0524 (8)	
H11	0.2690	0.5206	0.9677	0.063*	
C12	0.1173 (4)	0.6854 (3)	0.9054 (3)	0.0534 (8)	
H12	0.0117	0.6586	0.9557	0.064*	
C13	0.1186 (4)	0.8074 (3)	0.8242 (2)	0.0485 (7)	
H13	0.0134	0.8620	0.8190	0.058*	
C14	0.2180 (4)	0.9262 (3)	0.4043 (2)	0.0415 (6)	
C15	0.1925 (4)	0.8100 (3)	0.3660 (2)	0.0394 (6)	
C16	0.1873 (4)	0.6816 (3)	0.4374 (2)	0.0548 (8)	
H16	0.2020	0.6634	0.5147	0.066*	
C17	0.1604 (5)	0.5797 (3)	0.3946 (3)	0.0593 (8)	
H17	0.1600	0.4931	0.4423	0.071*	
C18	0.1345 (4)	0.6076 (3)	0.2821 (2)	0.0480 (7)	
C19	0.1422 (4)	0.7332 (3)	0.2085 (3)	0.0555 (8)	
H19	0.1275	0.7504	0.1312	0.067*	
C20	0.1724 (4)	0.8337 (3)	0.2515 (2)	0.0491 (7)	
H20	0.1792	0.9193	0.2021	0.059*	

### Atomic displacement parameters (Å^2^)

**Table:**

	*U*^11^	*U*^22^	*U*^33^	*U*^12^	*U*^13^	*U*^23^
Br1	0.0901 (3)	0.0740 (3)	0.0629 (2)	0.00901 (19)	−0.02581 (18)	−0.04231 (19)
Cl1	0.0828 (6)	0.0743 (6)	0.0825 (6)	−0.0160 (5)	−0.0070 (5)	−0.0520 (5)
O1	0.1015 (18)	0.0392 (12)	0.0457 (12)	−0.0063 (12)	−0.0087 (11)	−0.0094 (9)
O2	0.0902 (16)	0.0397 (12)	0.0469 (12)	−0.0099 (11)	−0.0193 (11)	−0.0075 (10)
N1	0.0639 (15)	0.0360 (13)	0.0407 (13)	−0.0063 (11)	−0.0108 (11)	−0.0149 (10)
N2	0.0763 (17)	0.0328 (13)	0.0384 (12)	−0.0087 (11)	−0.0132 (11)	−0.0115 (10)
C1	0.0478 (16)	0.0488 (18)	0.0520 (16)	0.0021 (13)	−0.0122 (13)	−0.0270 (14)
C2	0.0476 (16)	0.0391 (15)	0.0463 (16)	−0.0007 (12)	−0.0086 (13)	−0.0167 (13)
C3	0.0420 (15)	0.0330 (14)	0.0442 (15)	−0.0023 (11)	−0.0072 (12)	−0.0144 (12)
C4	0.0516 (17)	0.0392 (16)	0.0470 (17)	−0.0034 (13)	−0.0054 (13)	−0.0149 (13)
C5	0.068 (2)	0.0306 (15)	0.0629 (19)	−0.0036 (14)	−0.0084 (16)	−0.0122 (14)
C6	0.0555 (18)	0.0443 (18)	0.072 (2)	−0.0012 (14)	−0.0137 (15)	−0.0313 (16)
C7	0.0448 (15)	0.0379 (15)	0.0390 (15)	−0.0021 (12)	−0.0059 (12)	−0.0150 (12)
C8	0.0512 (16)	0.0305 (14)	0.0368 (14)	−0.0066 (12)	−0.0078 (12)	−0.0143 (11)
C9	0.0522 (18)	0.0433 (17)	0.0482 (16)	−0.0037 (14)	−0.0039 (13)	−0.0161 (13)
C10	0.064 (2)	0.0395 (17)	0.0618 (19)	0.0082 (15)	−0.0187 (17)	−0.0169 (15)
C11	0.077 (2)	0.0326 (16)	0.0472 (17)	−0.0099 (15)	−0.0152 (16)	−0.0066 (13)
C12	0.063 (2)	0.0460 (18)	0.0503 (17)	−0.0166 (16)	−0.0007 (15)	−0.0128 (14)
C13	0.0518 (18)	0.0431 (17)	0.0523 (17)	−0.0014 (13)	−0.0086 (14)	−0.0158 (14)
C14	0.0458 (16)	0.0412 (17)	0.0382 (15)	−0.0038 (12)	−0.0082 (12)	−0.0109 (13)
C15	0.0433 (15)	0.0388 (15)	0.0380 (14)	−0.0041 (12)	−0.0084 (12)	−0.0122 (12)
C16	0.083 (2)	0.0445 (17)	0.0403 (16)	−0.0140 (15)	−0.0156 (15)	−0.0112 (13)
C17	0.084 (2)	0.0423 (17)	0.0538 (19)	−0.0151 (16)	−0.0114 (17)	−0.0132 (14)
C18	0.0465 (16)	0.0549 (19)	0.0530 (17)	−0.0078 (14)	−0.0055 (13)	−0.0300 (15)
C19	0.069 (2)	0.064 (2)	0.0417 (16)	−0.0029 (16)	−0.0131 (14)	−0.0250 (15)
C20	0.0633 (19)	0.0458 (17)	0.0373 (15)	−0.0027 (14)	−0.0085 (13)	−0.0102 (13)

### Geometric parameters (Å, °)

**Table:**

Br1—C1	1.893 (3)	C8—C13	1.386 (4)
Cl1—C18	1.740 (3)	C9—C10	1.384 (4)
O1—C4	1.344 (3)	C9—H9	0.9300
O1—H1	0.8200	C10—C11	1.368 (4)
O2—C14	1.210 (3)	C10—H10	0.9300
N1—C7	1.285 (3)	C11—C12	1.372 (4)
N1—N2	1.370 (3)	C11—H11	0.9300
N2—C14	1.364 (3)	C12—C13	1.376 (4)
N2—H2	0.8600	C12—H12	0.9300
C1—C6	1.375 (4)	C13—H13	0.9300
C1—C2	1.380 (4)	C14—C15	1.491 (4)
C2—C3	1.390 (4)	C15—C20	1.378 (4)
C2—H2A	0.9300	C15—C16	1.382 (4)
C3—C4	1.407 (4)	C16—C17	1.385 (4)
C3—C7	1.476 (3)	C16—H16	0.9300
C4—C5	1.392 (4)	C17—C18	1.359 (4)
C5—C6	1.363 (4)	C17—H17	0.9300
C5—H5	0.9300	C18—C19	1.368 (4)
C6—H6	0.9300	C19—C20	1.381 (4)
C7—C8	1.493 (4)	C19—H19	0.9300
C8—C9	1.385 (4)	C20—H20	0.9300
			
C4—O1—H1	109.5	C11—C10—H10	119.8
C7—N1—N2	119.3 (2)	C9—C10—H10	119.8
C14—N2—N1	118.3 (2)	C10—C11—C12	119.8 (3)
C14—N2—H2	120.8	C10—C11—H11	120.1
N1—N2—H2	120.8	C12—C11—H11	120.1
C6—C1—C2	120.4 (3)	C11—C12—C13	120.4 (3)
C6—C1—Br1	119.3 (2)	C11—C12—H12	119.8
C2—C1—Br1	120.2 (2)	C13—C12—H12	119.8
C1—C2—C3	120.9 (3)	C12—C13—C8	120.4 (3)
C1—C2—H2A	119.6	C12—C13—H13	119.8
C3—C2—H2A	119.6	C8—C13—H13	119.8
C2—C3—C4	118.4 (2)	O2—C14—N2	121.2 (2)
C2—C3—C7	119.9 (2)	O2—C14—C15	122.5 (2)
C4—C3—C7	121.8 (2)	N2—C14—C15	116.3 (2)
O1—C4—C5	117.2 (2)	C20—C15—C16	118.5 (3)
O1—C4—C3	123.5 (2)	C20—C15—C14	117.1 (2)
C5—C4—C3	119.3 (3)	C16—C15—C14	124.4 (2)
C6—C5—C4	121.3 (3)	C15—C16—C17	120.5 (3)
C6—C5—H5	119.3	C15—C16—H16	119.8
C4—C5—H5	119.3	C17—C16—H16	119.8
C5—C6—C1	119.7 (3)	C18—C17—C16	119.3 (3)
C5—C6—H6	120.2	C18—C17—H17	120.4
C1—C6—H6	120.2	C16—C17—H17	120.4
N1—C7—C3	116.0 (2)	C17—C18—C19	121.8 (3)
N1—C7—C8	123.7 (2)	C17—C18—Cl1	118.7 (2)
C3—C7—C8	120.2 (2)	C19—C18—Cl1	119.5 (2)
C9—C8—C13	118.9 (2)	C18—C19—C20	118.4 (3)
C9—C8—C7	120.4 (2)	C18—C19—H19	120.8
C13—C8—C7	120.7 (2)	C20—C19—H19	120.8
C10—C9—C8	120.1 (3)	C15—C20—C19	121.5 (3)
C10—C9—H9	120.0	C15—C20—H20	119.2
C8—C9—H9	120.0	C19—C20—H20	119.2
C11—C10—C9	120.4 (3)		
			
C7—N1—N2—C14	178.9 (2)	C13—C8—C9—C10	−0.3 (4)
C6—C1—C2—C3	−1.2 (4)	C7—C8—C9—C10	179.2 (3)
Br1—C1—C2—C3	−179.6 (2)	C8—C9—C10—C11	−0.6 (4)
C1—C2—C3—C4	0.7 (4)	C9—C10—C11—C12	0.7 (4)
C1—C2—C3—C7	179.8 (2)	C10—C11—C12—C13	0.1 (4)
C2—C3—C4—O1	179.8 (3)	C11—C12—C13—C8	−1.0 (4)
C7—C3—C4—O1	0.8 (4)	C9—C8—C13—C12	1.1 (4)
C2—C3—C4—C5	0.2 (4)	C7—C8—C13—C12	−178.4 (3)
C7—C3—C4—C5	−178.9 (3)	N1—N2—C14—O2	0.0 (4)
O1—C4—C5—C6	179.7 (3)	N1—N2—C14—C15	179.8 (2)
C3—C4—C5—C6	−0.7 (4)	O2—C14—C15—C20	0.2 (4)
C4—C5—C6—C1	0.2 (5)	N2—C14—C15—C20	−179.6 (2)
C2—C1—C6—C5	0.7 (4)	O2—C14—C15—C16	−179.6 (3)
Br1—C1—C6—C5	179.2 (2)	N2—C14—C15—C16	0.5 (4)
N2—N1—C7—C3	180.0 (2)	C20—C15—C16—C17	−0.7 (5)
N2—N1—C7—C8	0.4 (4)	C14—C15—C16—C17	179.1 (3)
C2—C3—C7—N1	178.4 (2)	C15—C16—C17—C18	−1.6 (5)
C4—C3—C7—N1	−2.5 (4)	C16—C17—C18—C19	2.9 (5)
C2—C3—C7—C8	−2.0 (4)	C16—C17—C18—Cl1	−175.6 (2)
C4—C3—C7—C8	177.1 (2)	C17—C18—C19—C20	−1.7 (5)
N1—C7—C8—C9	79.9 (3)	Cl1—C18—C19—C20	176.7 (2)
C3—C7—C8—C9	−99.7 (3)	C16—C15—C20—C19	1.9 (4)
N1—C7—C8—C13	−100.6 (3)	C14—C15—C20—C19	−177.9 (3)
C3—C7—C8—C13	79.9 (3)	C18—C19—C20—C15	−0.7 (5)

### Hydrogen-bond geometry (Å, °)

**Table:**

*D*—H···*A*	*D*—H	H···*A*	*D*···*A*	*D*—H···*A*
O1—H1···N1	0.82	1.84	2.554 (3)	145

## References

[bb1] Bruker (1996). *SMART* and *SAINT* Bruker AXS Inc., Madison, Wisconsin, USA.

[bb2] Carcelli, M., Mazza, P., Pelizzi, G. & Zani, F. (1995). *J. Inorg. Biochem.* **57**, 43–62. 10.1016/0162-0134(94)00004-t 7876834

[bb3] Salem, A. A. (1998). *Microchem. J.* **60**, 51–66.

[bb4] Sheldrick, G. M. (1996). *SADABS* University of Göttingen, Germany.

[bb5] Sheldrick, G. M. (2008). *Acta Cryst.* A**64**, 112–122. 10.1107/S0108767307043930 18156677

[bb6] Singh, R. B., Jain, P. & Singh, R. P. (1982). *Talanta*, **29**, 77–84. 10.1016/0039-9140(82)80024-6 18963087

